# Gene copy number and negative feedback differentially regulate transcriptional variability of segmentation clock genes

**DOI:** 10.1016/j.isci.2022.104579

**Published:** 2022-06-11

**Authors:** Oriana Q.H. Zinani, Kemal Keseroğlu, Supravat Dey, Ahmet Ay, Abhyudai Singh, Ertuğrul M. Özbudak

**Affiliations:** 1 Department of Pediatrics, University of Cincinnati College of Medicine, Cincinnati, OH 45229, USA; 2 Division of Developmental Biology, Cincinnati Children’s Hospital Medical Center, Cincinnati, OH 45229, USA; 3 Department of Electrical and Computer Engineering, Biomedical Engineering and Mathematical Sciences, University of Delaware, Newark, DE 19716, USA; 4 Departments of Biology and Mathematics, Colgate University, Hamilton, NY 13346, USA

**Keywords:** Biological sciences, Chronobiology, Developmental biology

## Abstract

Timely progression of a genetic program is critical for embryonic development. However, gene expression involves inevitable fluctuations in biochemical reactions leading to substantial cell-to-cell variability (gene expression noise). One of the important questions in developmental biology is how pattern formation is reproducibly executed despite these unavoidable fluctuations in gene expression. Here, we studied the transcriptional variability of two paired zebrafish segmentation clock genes (*her1* and *her7*) in multiple genetic backgrounds. Segmentation clock genes establish an oscillating self-regulatory system, presenting a challenging yet beautiful system in studying control of transcription variability. In this study, we found that a negative feedback loop established by the Her1 and Her7 proteins minimizes uncorrelated variability whereas gene copy number affects variability of both RNAs in a similar manner (correlated variability). We anticipate that these findings will help analyze the precision of other natural clocks and inspire the ideas for engineering precise synthetic clocks in tissue engineering.

## Highlights

• Cell volume variability causes correlated transcriptional variability of clock genes
• Her1/7 negative feedback loop suppresses uncorrelated transcriptional variability
• Gene dosage impacts correlated transcriptional variability of segmentation clock genes

## Graphical abstract

**Figure:**
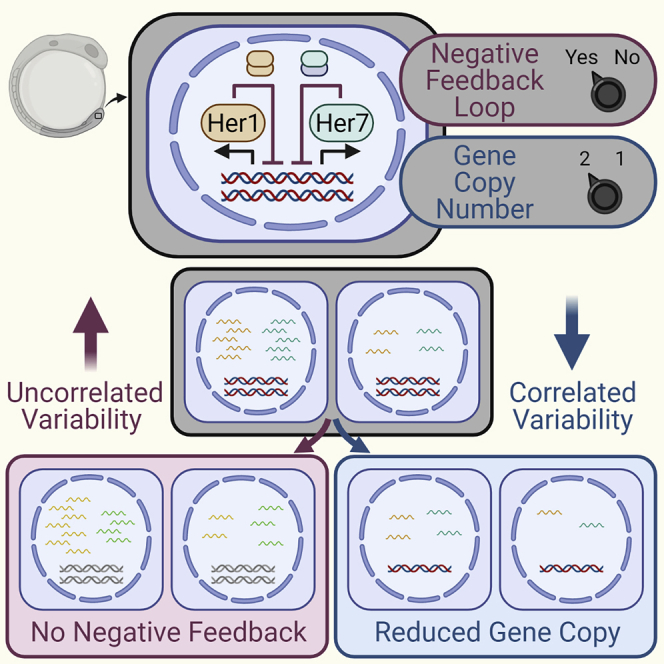

## Introduction

**Figure 1:**
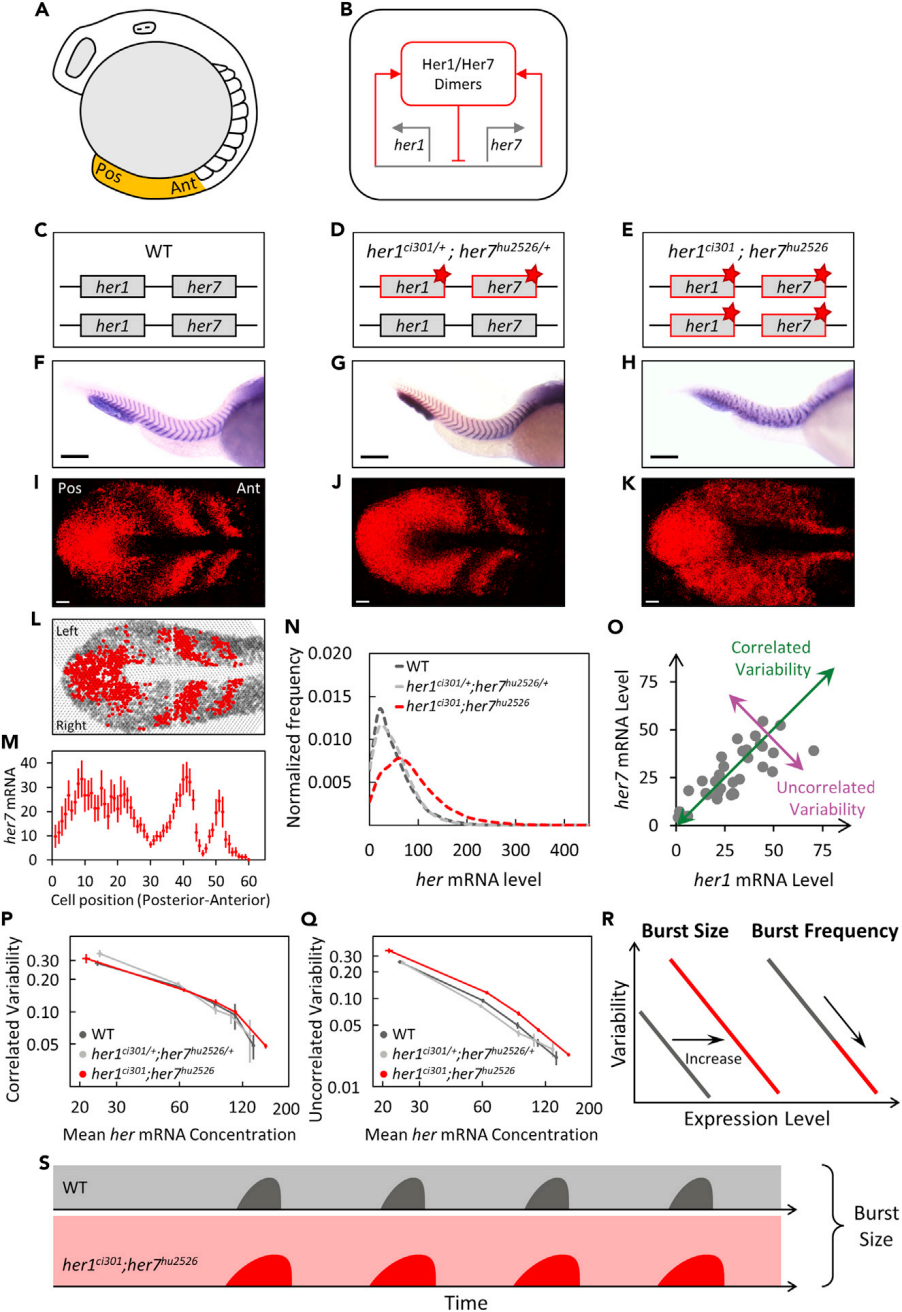
Negative feedback loop established by Her1/7 proteins suppresses uncorrelated transcriptional variability (A) The sketch of a wild-type zebrafish embryo at the 12-somite stage. The PSM, highlighted in orange, is dissected from the embryo for confocal imaging. Anterior (Ant) is to the right, and posterior (Pos) is to the left. (B) Her1 and Her7 repress transcription of their own genes, and thereby form a cell-autonomous negative feedback loop. (C–E) Red stars (∗) mark *her1* and *her7* genes carrying point mutations causing premature stop codons. (F–H) The boundaries of somite segments are marked by *xirp2* ISH staining in wild-type (F), *her1*^*ci301/+*^;*her7*^*hu2526/+*^ (G), and *her1*^*ci301*^;*her7*^*hu2526*^ mutants (H). Scale bar is 200 μm. (I-J) Expression of *her7* displays kinematic waves and oscillations in wild-type (I) and *her1*^*ci301/+*^;*her7*^*hu2526/+*^ (J) mutants. (K) Expression pattern of *her7* is disrupted in double homozygous *her1*^*ci301*^;*her7*^*hu2526*^ mutants. Scale bar is 30 μm. (L) The PSM is divided into single-cell width slices. Red or gray circles represent the cells, which contain higher or lower RNA levels than an arbitrary threshold, respectively. Three oscillatory waves of *her7* are visible. The images are divided into two portions as left (top) and right (bottom) halves of the PSM. (M) *her7* RNA counts are plotted along the right half of PSM (posterior-to-anterior). Each dot corresponds to the mean RNA number in a sliced cell population. Error bars are 2 SEM. (N) The frequency histogram of total *her* (*her1*+*her7*) RNA per cell is plotted in wild-type (dark gray) (n*=24*, *N=2*), *her1*^*ci301/+*^;*her7*^*hu2526/+*^ (silver) (n*=18*, *N=2*), *her1*^*ci301*^;*her7*^*hu2526*^ (red) (n*=28*, *N=2*) mutant embryos. (O) Each dot represents *her1* (x axis) and *her7* (y axis) counts in a single cell. Correlated (green) and uncorrelated (purple) variability of *her* expression can be quantified in the population. (P and Q) Correlated (P) and uncorrelated (Q) transcriptional variability of *her1* and *her7* are plotted based on mean *her* RNA concentration (mRNA counts normalized by cell volume). Error bars are two standard errors. (R) Changing the size or frequency of transcriptional bursts affect the variability curve (CV^2^ vs. mean) differently. (S) Loss of Her proteins results in more uncorrelated transcriptional variability most likely due to increased burst sizes in *her1*^*ci301*^;*her7*^*hu2526*^ mutants. *n* is the number of embryos; *N* is the number of independent experiments. See also Figure S1.

Gene expression is an inherently stochastic process because of diffusion-driven biochemical processes involving small numbers of molecules (Elowitz et al., 2002; Ozbudak et al., 2002). Many developmental processes, such as pattern formation, are dependent upon coordinated expression of key genes. Somitogenesis is a landmark example of developmental pattern formation, generating metameric organization of the major body axis in vertebrates (Hubaud and Pourquie, 2014). Somites contain the precursor cells of the musculoskeletal system. Sequential segmentation of somites continues for a species-specific number (e.g., 33 times in zebrafish) until patterning of the full body axis is completed. During somitogenesis, groups of cells periodically form a somite segment (e.g., ∼200 cells form a somite every 30 min in zebrafish). The period of segmentation is controlled by the oscillatory expression of segmentation clock genes in the presomitic mesoderm (PSM) (Figure 1A) (Hubaud and Pourquie, 2014). *Hes/her* family genes form the core of the segmentation clock: their expression oscillate in vertebrate embryos and disrupting their oscillations leads to vertebral segmentation defects in animal models and congenital scoliosis (i.e., spondylocostal dysplasia) in patients (Hubaud and Pourquie, 2014). Given the rapid tempo and reproducible precision of segmentation, variability of clock expression should be tightly regulated.

In zebrafish, two linked genes—*her1* and *her7—*have been identified as central to the genesis of oscillations (Figure 1B). When both are deleted (Henry et al., 2002) or mutated (Lleras Forero et al., 2018; Zinani et al., 2021), all signs of oscillation are lost and segment boundary formation is disrupted along the body axis (Figures 1C–1K). Oscillations are generated by a transcriptional negative feedback loop (Ay et al., 2013; Giudicelli et al., 2007; Harima et al., 2013; Lewis, 2003; Schroter et al., 2012). Her1 and Her7 form different types of dimers that repress their own transcription (Ay et al., 2013; Schroter et al., 2012; Trofka et al., 2012). This negative feedback loop drives oscillatory expression of both *her1* and *her7*. Because *her1* and *her7* have similar RNA half-lives (Giudicelli et al., 2007) and transcriptional time delays (Hanisch et al., 2013), transcript levels of both genes are very similar (Keskin et al., 2018; Zinani et al., 2021). Owing to the negative feedback loop, clock RNAs are both the input and output of the clock proteins, and thereby their variability is a good proxy for the function of the segmentation clock.

The oscillation period of segmentation clock genes increases incrementally along the posterior-to-anterior (tail-to-head) direction in the PSM (Giudicelli et al., 2007; Gomez et al., 2008). This slowing down of oscillations causes a phase delay between the cells located in the anterior and posterior PSM. As a consequence, it leads to different phases of the oscillator cycle in space along the PSM and two to three kinematic waves of gene expression in the oscillation cycle at any moment (Figures 1I, 1L, and 1M). Hence, all cells located at the same posterior-anterior position in a two-dimensional, single-cell-wide cross-section are in the same phase of oscillations. To quantify transcriptional variability of *her1* and *her7*, we exploited this unique spatial property of the segmentation clock. To group cells in the same oscillation phase, we grouped cells in the same spatial location (Figures 1L and 1M).

To study changes in clock gene expression during zebrafish somitogenesis, we recently performed high-resolution single-molecule fluorescence *in situ* hybridization (smFISH) to count mRNA transcripts in single cells (Keskin et al., 2018). We then quantified mean and variability (CV^2^ [SD/mean]^2^) of transcript levels among phase-grouped cell populations (i.e., single-cell-diameter slices). Because transcriptional variability will depend on RNA levels, we then grouped variability data into five bins based on mean RNA levels. We found that segmentation clock genes are transcribed at low levels (mean of total *her1* plus *her7* RNA is 49 molecules) (Figure 1N) and display high variability (CV^2^ ranges from 0.15 to 0.60 at different expression levels) (Keskin et al., 2018; Zinani et al., 2021). We further showed that correlated variability contributes more to total transcriptional variability than uncorrelated variability (73% vs. 27%, p < 0.001, Figures S1A and 1O).

To generate fast oscillations in zebrafish, segmentation clock RNAs and proteins have extremely short half-lives (t_1/2_ = 3–5 min) (Ay et al., 2013; Giudicelli et al., 2007). Thus, variability in their levels cannot be reduced by simple temporal averaging, which causes the segmentation clock to be very noisy (Keskin et al., 2018). We recently showed that pairing of *her1* and *her7* on the same chromosome promotes their correlated expression to ensure proper development (Zinani et al., 2021). However, the source of high transcriptional variability of the segmentation clock genes is yet to be determined (Keskin et al., 2018).

In this study, we explicitly investigated the impact of three factors on the transcriptional variability of *her1* and *her7*: (1) variation of cell volume and its associated resources, (2) negative feedback by Her1 and Her7 transcriptional repressors, and (3) gene dosage. We found that all three factors differentially contribute to transcriptional variability.

## Results

### Cell volume dependent factors increase correlated transcriptional variability of clock genes

Cell volume is a general regulator of gene expression through its ubiquitous effect on molecular concentrations (Song et al., 2015). It was previously shown that transcriptional burst size correlates with cell volume (Padovan-Merhar et al., 2015). We assessed whether variation in cell volume could underlie transcriptional variability of clock genes by normalizing transcript counts by cell volume (Keskin et al., 2018). This analysis revealed that uncorrelated variability does not depend on cell volume (Figure S1B), but roughly 38% of correlated variability can be filtered out by converting RNA numbers to concentrations (Figure S1C). These results show that variability in volume-dependent factors increases the dominant correlated transcriptional variability. We believe the increased correlated variability is likely triggered by transcriptional cofiring of *her1* and *her7* occurring independently in different cells. Nonetheless, a large portion of variability remained to be explained (from now on, volume-corrected variability is plotted in all figures).

### Negative feedback loop suppresses uncorrelated transcriptional variability of clock genes

What is the source of the size-independent correlated variability? One possibility is cell to cell variability in the levels of an upstream transcriptional regulator (e.g., Her1 and Her7), which could cause high transcriptional covariation of target genes (i.e., noise transmission). Indeed, a theoretical study previously proposed that slow dissociation of Her1/7 repressors from DNA is the main factor causing transcriptional variability of clock genes (Jenkins et al., 2015). According to this model, abolishing the function of repressors should significantly decrease transcriptional variability of clock genes. To test this hypothesis and discern the role of negative feedback loop in controlling transcriptional variability, we reanalyzed smFISH data obtained in *her1*^*ci301*^;*her7*^*hu2526*^ double homozygous mutants (Zinani et al., 2021). Unlike wild-type and heterozygote mutant embryos, oscillatory expression of clock genes is lost, and segmentation is disrupted in double homozygous mutants. Because our probes do not distinguish between mutant and wild-type RNAs, we found that heterozygous mutants have similar RNA levels compared to wild-type embryos. In contrast, the mean RNA level increased by 74% (p < 0.001) in homozygous mutants compared to wild-type embryos (Zinani et al., 2021) (Figure 1N). Because transcriptional variability depends on mean RNA levels (Figure S1A), we compared variability of mutant and wild-type embryos at similar mean RNA levels. The transcriptional variability of the clock genes was similar in wild-type and double heterozygous mutants (Figures 1P and 1Q, 5% difference for correlated variability, 8% difference for uncorrelated variability). Opposite to the previously proposed hypothesis (Jenkins et al., 2015), the transcriptional variability is higher rather than lower in double homozygous mutants compared to wild-type embryos: although the correlated variability modestly increased by 4.6% (Figure 1P, p < 0.001), the uncorrelated variability of clock genes increased by 33% (Figure 1Q, p < 0.001). On the other hand, these results are consistent with an earlier report that negative feedback reduces variability of non-oscillating synthetic reporters in bacteria (Becskei and Serrano, 2000). According to the two-state transcriptional bursting models, the variability curve (CV^2^ vs. mean) can only be uplifted by increasing the size rather than frequency of transcriptional bursts (Figure 1R) (Dar et al., 2012). Therefore, these findings suggest that Her1/7 might suppress burst sizes to decrease the transcriptional variability of clock genes (Figure 1S). In conclusion, Her1/7 repressors do not increase clock gene transcriptional variability, as previously proposed (Jenkins et al., 2015), instead they participate in a cell-autonomous negative feedback loop, which decreases uncorrelated variability.

### Gene dosage increases correlated transcriptional variability of clock genes

**Figure 2:**
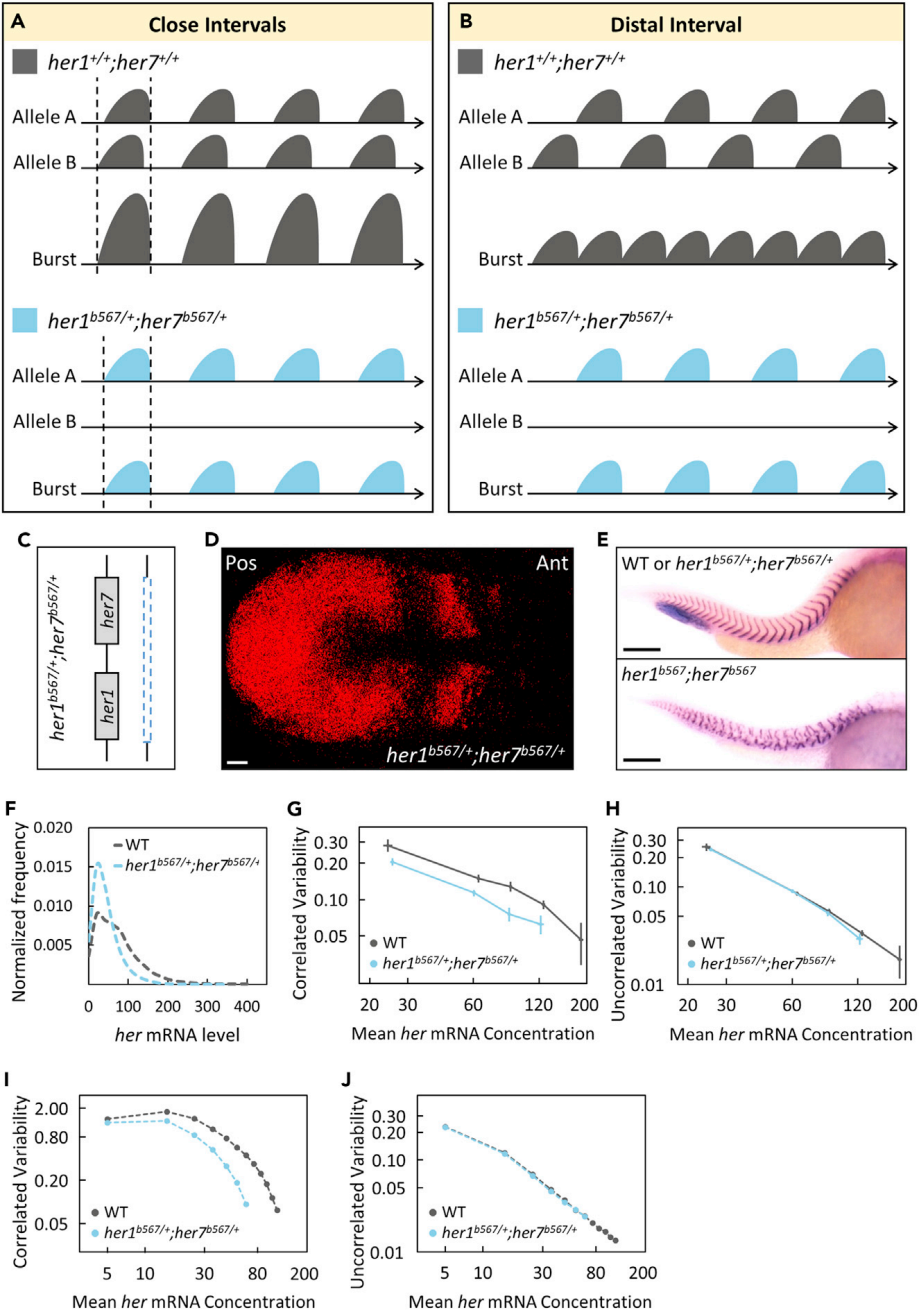
Clock gene dosage increases correlated transcriptional variability (A and B) Reduced gene copy in *her1*^*b567/+*^;*her7*^*b567/+*^ mutants results in decreased burst sizes or frequency if two homologous alleles cofire with either close (A) or distal (B) interval, respectively. (C) One of the chromosomes has a large deletion including the *her1*-*her7* locus in *her1*^*b567/+*^;*her7*^*b567/+*^ mutants. (D) A *her1*^*b567/+*^;*her7*^*b567/+*^ embryo with normal kinematic waves of *her7* transcription. Scale bar is 30 μm. (E) The boundaries of somite segments are marked by *xirp2* ISH staining in sibling (top) wild-type or heterozygous *her1*^*b567/+*^;*her7*^*b567/+*^ and (bottom) homozygous *her1*^*b567*^;*her7*^*b567*^ mutant embryos. Scale bar is 200 μm. (F) *her1*^*b567/+*^;*her7*^*b567/+*^ embryos (n*=24*, *N=2*) have less *her* mRNA than wild-type (n*=14*, *N=2*). The frequency histogram of total *her* (*her1*+*her7*) RNA per cell is plotted in wild-type (dark gray), *her1*^*b567/+*^;*her7*^*b567/+*^ mutant (light blue) embryos. (G and H) *her1*^*b567/+*^;*her7*^*b567/+*^ embryos have reduced correlated transcriptional variability than wild-type (G), but uncorrelated transcriptional variability changed mildly (H). Reduced correlated transcriptional variability can be explained by reduced burst size in *her1*^*b567/+*^;*her7*^*b567/+*^ embryos than in wild-type. (I and J) Computational model also shows more reduced correlated variability (I) in *her1*^*b567/+*^;*her7*^*b567/+*^ embryos than in wild-type embryos compared to uncorrelated variability (J). Error bars are two standard errors. *n* is the number of embryos; *N* is the number of independent experiments. See also Tables S1–S3.

We next investigated the role of gene dosage on the transcriptional variability of clock genes. If transcription of clock genes has large bursts, it could influence transcriptional variability by two alternative scenarios: (1) if two chromosomes fire at close time intervals (Figure 2A), overlapping bursts will increase burst sizes and thereby uplift variability curve (Figure 1R); (2) if chromosomes fire at distant time intervals (Figure 2B), nonoverlapping bursts will increase burst frequency and this will shift the variability curve only diagonally (Figure 1R).

Gene copy numbers could be changed by utilizing a chromosomal deletion mutant covering the entire *her1-her7* locus (Henry et al., 2002). These heterozygous chromosomal deletion embryos have normal clock expression pattern in PSM, and no somite segmentation defect (Henry et al., 2002) (Figures 2C–2E). We previously reported that the levels and spatial amplitudes of the clock RNAs were lower in the *her1*^*b567/+*^;*her7*^*b567/+*^ chromosomal deletion mutants compared to wild-type embryos (28% *her1* amplitude p value = 0.017, 28% *her7* amplitude p < 0.001 (Zinani et al., 2021), and 38% total *her* level p < 0.001, Figure 2F). In contrast to our findings using the *her1*^*ci301/+*^;*her7*^*hu2526/+*^ heterozygous mutants (Figures 1P and 1Q), we found that correlated transcriptional variability decreased 31% (p < 0.001), whereas uncorrelated transcriptional variability changed only 4.5% in *her1*^*b567/+*^;*her7*^*b567/+*^ mutants compared to wild-type embryos (Figures 2G and 2H). The variability curve can only be shifted down by decreasing transcriptional burst size (Figure 1R) (Dar et al., 2012). Therefore, our results suggest that decreased gene copy number primarily decreases transcriptional burst size. Our current results support the scenario that both chromosomes fire at close time intervals, and are in agreement with our previous findings reporting around 60% cofiring of two homologous alleles (Zinani et al., 2021).

Decreased bursting alone cannot explain all of our results, because high burst sizes generally increase uncorrelated transcriptional variability (Raj et al., 2006) instead of correlated variability that we measured (Figures 2G and 2H). However, chromosomal adjacency was shown to cause correlated variability of synthetic reporters in yeast, mammalian cell culture, and fly embryos (Becskei et al., 2005; Fukaya et al., 2016; Raj et al., 2006). We recently showed that pairing of two clock genes on the same chromosome drives their transcriptional cofiring (Zinani et al., 2021). Therefore, we conclude that transcriptional cofiring leads to high correlated transcriptional variability in wild-type embryos and reducing gene copy numbers primarily decreases correlated variability.

**Figure 3:**
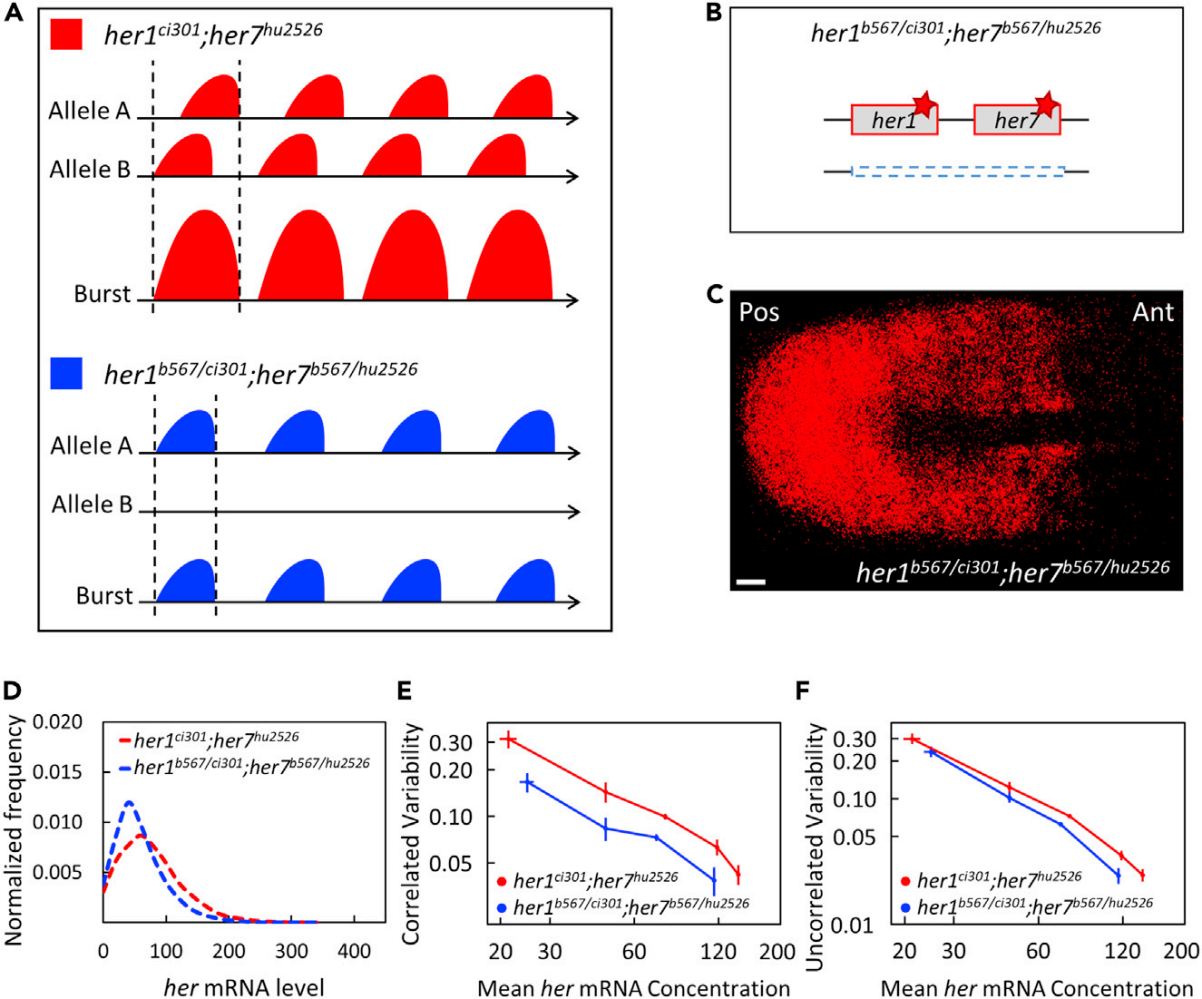
Clock gene dosage increases correlated transcriptional variability more than uncorrelated one (A) Reduced copy of *her* genes likely results in decreased burst sizes in *her1*^*b567/ci301*^;*her7*^*b567/hu2526*^ mutants compared to *her1*^*ci301*^;*her7*^*hu2526*^ mutants. (B) One of the chromosomes has mutant *her1* and *her7* genes whereas the other has a large deletion including the *her1*-*her7* locus in *her1*^*b567/ci301*^;*her7*^*b567/hu2526*^ mutants. Red stars (∗) mark point mutations causing premature stop codons. (C) Expression pattern of *her7* is disrupted in *her1*^*b567/ci301*^;*her7*^*b567/hu2526*^ embryos. Scale bar is 30 μm. (D) *her1*^*b567/ci301*^;*her7*^*b567/hu2526*^ embryos (n*=12*, *N=2*) have less *her* mRNA than double homozygous *her1*^*ci301*^;*her7*^*hu2526*^ embryos (n*=17*, *N=2*). The frequency histogram of total mutant *her* (*her1*+*her7*) RNA per cell is plotted in *her1*^*ci301*^;*her7*^*hu2526*^ (red), *her1*^*b567/ci301*^;*her7*^*b567/hu2526*^ mutant (blue) embryos. (E and F) *her1*^*b567/ci301*^;*her7*^*b567/hu2526*^ embryos have both reduced correlated (E) and uncorrelated (F) transcriptional variability than *her1*^ci301^;*her7*^*hu2526*^. Error bars are two standard errors. *n* is the number of embryos; *N* is the number of independent experiments.

We inferred potential changes of transcriptional bursts by comparing heterozygous chromosomal deletion mutants with wild-type embryos. To further assess the role of gene dosage on transcriptional variability, we took two additional approaches. Firstly, we leveraged a simple model of bursty transcription. Simulations showed that reducing gene dosage in an oscillating system changed correlated variability more than uncorrected variability of the clock genes (Figures 2I and 2J). These simulations supported our experimental results. Secondly, we tested the gene dosage effect in a non-oscillating system by generating two different double homozygous mutants (Figure 3A): The first double homozygous mutant, *her1*^*ci301*^;*her7*^*hu2526*^, carries mutants of both genes on two chromosomes, whereas the second one *her1*^*b567/ci301*^;*her7*^*b567/hu2526*^ carries mutant genes only on one chromosome (both genes are deleted in the homologous chromosome) (Figure 3B). We performed smFISH experiments (Figure 3C) and found that the levels of clock RNAs were 23% lower in *her1*^*b567/ci301*^;*her7*^*b567/hu2526*^ compared to *her1*^*ci301*^;*her7*^*hu2526*^ mutants (p < 0.001, Figure 3D). We found that correlated transcriptional variability decreased 38% (p < 0.001) while uncorrelated transcriptional variability decreased only 18% (p < 0.001) in *her1*^*b567/ci301*^;*her7*^*b567/hu2526*^ compared to *her1*^*ci301*^;*her7*^*hu2526*^ mutants (Figures 3E and 3F). These results validated our conclusions and showed that reducing gene copy numbers primarily decreases correlated variability.

## Discussion

Our previous study showed that correlated variability contributes more to total transcriptional variability than uncorrelated variability (Keskin et al., 2018); however, the source of the transcription variability was unknown. In this study, we combined single-cell transcript counting with genetic manipulations to investigate the roles of cell volume, transcriptional negative feedback loop, and gene dosage on transcriptional variability of two segmentation clock genes. We found that Her1/7 cell-autonomous negative feedback loop decreases uncorrelated transcriptional variability likely by reducing transcriptional burst size. In contrast, cell volume and gene dosage increased the correlated transcriptional variability of clock genes. Our results support a model where highly correlated variability of two clock genes is likely because of coordinated transcriptional bursting between two genes on two homologous alleles. Correlated variability of *her1* and *her7* expression can be influenced by (i) gene-dosage, (ii) volume-dependent, and (iii) volume-independent cellular factors. Our results support that adjacency of two segmentation clock genes causes their correlated expression, a phenomenon previously shown to be beneficial for robust developmental pattern formation in zebrafish embryos (Zinani et al., 2021).

The levels of clock RNAs were 38% lower in the *her1*^*b567/+*^;*her7*^*b567/+*^ chromosomal deletion mutants compared to that in wild-type embryos (p < 0.001, Figure 2F). This lack of compensation in the expression levels suggests that the segmentation network differs from dosage compensated networks, such as the GAL network in yeast (Peng et al., 2016). In contrast, the functional outcome, (i.e., somite segmentation) can be successfully achieved at most of the physiological developmental temperatures (Figure 2E), suggesting phenotypic compensation might occur at a different regulatory step. On the other hand, both zebrafish segmentation and yeast GAL networks minimize noise in part by utilizing a negative feedback loop.

Oscillations are prevalent in biological systems, and Hes/Her protein levels oscillate in multiple cell types and tissues and control proliferation to differentiation switches (Kobayashi and Kageyama, 2014). We anticipate our findings would aid in understanding the precision of other natural oscillators and engineering precise synthetic oscillators. Our findings might inspire future studies for developing new ways to control stem cell proliferation and differentiation by engineering synthetic clocks or manipulating natural ones in tissue engineering or organoid systems.

## Limitations of the study

In this study, we used static smFISH experiments to identify the roles of transcriptional feedback loop and gene dosage on transcriptional variability of segmentation clock genes. We also performed stochastic simulations of transcriptional dynamics by using a simple model. From our results, we inferred that transcription of segmentation clock genes is bursty. However, direct demonstration of transcriptional bursts requires live imaging of RNA transcription. Future RNA live imaging experiments will shed light on the dynamics of transcriptional bursts, i.e., burst sizes and the frequency of bursts in a single clock cycle.

## STAR★Methods

### Key resources table

**Table:** 

REAGENT or RESOURCE	SOURCE	IDENTIFIER
**Antibodies**		

Chicken IgY, anti-GFP, unconjugated, Primary Antibody	Thermo Fisher Scientific	Cat#A10262, RRID: AB_2534023
Alexa Fluor 488 Goat anti-Chicken IgG (H+L) Secondary Antibody	Thermo Fisher Scientific	Cat#A-11039; RRID: AB_142924

**Chemicals, peptides, and recombinant proteins**		

RNAscope Fluorescent Multiplex Detection Reagents	Advanced Cell Diagnostics	Cat#320851
RNAscope Protease III Reagents	Advanced Cell Diagnostics	Cat#322340
Hoechst trihydrochloride, trihydrate	Invitrogen	Cat#33342
ProLong Gold antifade reagent	Life Technologies	Cat#P36934
SP6 mMessage mMachine	Life Technologies	Cat#AM1340
RNAscope Probe - Dr-her1-LE2-C3	Advanced Cell Diagnostics	Cat#433201-C3
RNAscope Probe - Dr-her7	Advanced Cell Diagnostics	Cat#428611

**Deposited data**		

Image Processing Pipeline	(Keskin et al., 2018)	Data S1 in (Keskin et al., 2018)
Stochastic Simulations Script	This paper	Data S1
Excel file of smFISH data for *her1*^*ci301*^;*her7*^*hu2526*^ embryos.	This paper	Data S2
Excel file of smFISH data for *her1*^*b567/ci301*^;*her7*^*b567/hu2526*^ embryos.	This paper	Data S3
Raw and analyzed data related to Figures 1 and 2	(Zinani et al., 2021)	https://www.ebi.ac.uk/biostudies/studies/S-BSST434
Raw data related to Figure 3	This paper	https://www.ebi.ac.uk/biostudies/studies/S-BSST847

**Experimental models: Organisms/strains**		

Zebrafish: *her1*^*ci301*^;*her7*^*hu2526*^	(Zinani et al., 2021)	ZFIN ID: ZDB-ALT-211025-4
Zebrafish: Df(Chr05:her1,her7,ndrg3a)b567	(Henry et al., 2002)	ZFIN ID: ZDB-ALT-030512-2

**Software and algorithms**		

Imaris 9.8	Bitplane	http://www.bitplane.com/imaris/imaris; RRID:SCR_007370
Python Programming Language, version 3.8	Python Software Foundation	http://www.python.org/; RRID:SCR_008394
Matlab_R2020b	Mathworks	http://www.mathworks.com/products/matlab/; RRID:SCR_001622
ImageJ		https://imagej.nih.gov/ij/; RRID:SCR_003070
GraphPad Prism 7	GraphPad	http://www.graphpad.com/; RRID:SCR_002798

**Other**		

Nikon A1R GaAsP inverted confocal microscope 100× 1.49 NA Apo TIRF DIC- Oil objective	Nikon	N/A

### Resource availability

#### Lead contact

• Further information and requests for resources and reagents should be directed to the lead and corresponding author Ertuğrul M. Özbudak (Ertugrul.Ozbudak@cchmc.org).

#### Materials availability

• This study did not generate new unique reagents.

### Experimental model and subject details

#### Fish stocks

Df(Chr05:her1,her7,ndrg3a)b567 (Henry et al., 2002) and *her1*^*ci301*^;*her7*^*hu2526*^ (Zinani et al., 2021) mutant lines were used in this study. The fish experiments were performed under the ethical guideline of Cincinnati Children’s Hospital Medical Center. The animal protocol was reviewed and approved by Cincinnati Children’s Hospital Medical Center Animal Care and Use Committees (Protocol # 2020-0031). Sex is not determined chromosomally and it is fixed weeks after fertilization in zebrafish. We used embryos less than one day post fertilization. Thus, we did not discriminate against a particular gender in our studies.

### Method details

#### smFISH and imaging

The smFISH experiments and confocal imaging were performed as described in (Zinani et al., 2021). Background subtracted total *her (her1+her7)* mRNA levels were plotted as RNA distributions. Transcriptional variability was plotted from background subtracted and volume corrected *her* levels for each genetic background as in (Keskin et al., 2018).

### Quantification and statistical analysis

#### Calculating transcriptional variability

By using previously published smFISH data (Zinani et al., 2021), we quantified transcription variability among cells located in single-cell-wide cross-sections along the PSM. Correlated, uncorrelated and total transcriptional variability were computed for cells located in each slice using the following equations as in (Keskin et al., 2018):

$$\text{uncorrelated}\;\text{variability}=\;\frac{1}{2}\left\langle {\left(\frac{her1}{\left\langle her1\right\rangle }-\frac{her7}{\left\langle her7\right\rangle }\right)}^{2}\right\rangle$$

$$\text{correlated}\;\text{variability}=\;\frac{\left\langle her1\;\cdot her7\right\rangle -\left\langle her1\right\rangle \left\langle her7\right\rangle }{\left\langle her1\right\rangle \left\langle her7\right\rangle }$$

totalvariability=uncorrelatedvariability+correlatedvariability

Transcriptional variability was plotted at different mean *her* (*her1+her7*) mRNA levels for each genetic background. We have previously showed how transcriptional variability varies with respect to mean *her* mRNA levels among phase grouped cells (single-cell diameter spatial slices) in wild-type embryos (Keskin et al., 2018). Then, we have grouped the data of individual slices into 5 bins according to their mean *her* mRNA numbers (Figure S1A) as in (Keskin et al., 2018).

#### Statistical analysis

To compare the correlated/uncorrelated variability in different genetic backgrounds we sampled the variability data with replacement 100 times, and for each case calculated the area under the variability curve (AUC). Then we assessed the statistical significance of the difference in the variability between different genetic backgrounds with paired t-test using the 100 AUC values. Normality was assessed Shapiro-Wilk test, and by visual inspection of histograms and normal Q-Q plots. The distributions of mRNA levels across different genetic backgrounds are compared by visual inspection of box-plots and the Kolmogorov-Smirnov test.

#### Computational modeling

To gain a better understanding of the noise properties of the segmentation clock in zebrafish, we adapted and modified the simple model proposed by Lewis (2003). Her1 and Her7 proteins form a heterodimer that inhibits their own expression. In our model, *her1* and *her7* mRNA production occurs in transcriptional bursts. We assume that heterodimer-induced repression has a direct effect on the average burst size of the mRNAs, but not their burst frequency. Additionally, we assume that *her1* and *her7* mRNA bursts are correlated. In our model, the time delays are implemented through 10 intermediate reactions. Below, we discuss the model in detail.

All molecular species in the model are summarized in Table S1, and the biochemical reactions are summarized in Table S2. The parameters ${\text{k}}_{\text{m}1}$, and ${\text{k}}_{\text{m}7}$ are burst frequencies for individual bursts of *her1*, and *her7* mRNAs, whereas ${\text{k}}_{\text{m}17}$ is the burst frequency of correlated bursts, i.e., when both genes fire together. We characterize the correlated bursts by the parameter α: ${\text{k}}_{\text{m}1}={\text{k}}_{\text{m}7}={\text{k}}_{\text{m}}\left(1-\text{α}\right)$, and ${\text{k}}_{\text{m}17}={\text{k}}_{\text{m}}\text{α}$. If α = 1, the bursts are perfectly correlated, and α=0 they are uncorrelated. The heterodimer (p17) repress the burst size. Let ${\text{B}}_{\text{max}}$ be the maximum burst size. In the presence of feedback, the average burst size is given by,

$$\left\langle \text{B}\right\rangle ={\text{B}}_{\text{max}}\text{f}\left(\text{p}17\right).$$

Here the repression function, f(p17), is the usual Hill function

$$\text{f}\left(\text{p}17\right)=\frac{1}{1+{\left(\frac{\text{p}17}{{\text{pd}}_{\text{crit}}}\right)}^{2}},$$

where ${\text{pd}}_{\text{crit}}$ is the amount of heterodimer counts at which the repression is half of the maximum. When ⟨B⟩≥1, we draw burst size from a geometric distribution: $\text{prob}\left(\text{B}=\text{i}\right)={\left(1-\text{s}\right)}^{\text{i}-1}\text{s},\;\text{for}\;\text{i}=1,2,3,\ldots ,$ where $\frac{1}{\text{s}}=\left\langle B\right\rangle$ is the average burst size.

The symbols ${\text{τ}}_{\text{m}}$, and ${\text{τ}}_{\text{p}}$ represent the transcriptional and translational delays. We incorporated these delays via n intermediate molecules for each mRNA and protein species. ${\text{k}}_{\text{p}1}$ and ${\text{k}}_{\text{p}7}$ are the rates for production of Her1 and Her7 proteins. The degradation rates for *her1* and *her7* mRNAs and proteins are denoted as ${\text{γ}}_{\text{m}1},{\text{γ}}_{\text{m}7},{\text{γ}}_{\text{p}1},$ and ${\text{γ}}_{\text{p}7}$. Her1 and Her7 proteins bind with rate ${\text{k}}_{\text{b}}$ to form heterodimer and they dissociate with rate ${\text{k}}_{\text{u}}$. The degradation rate of the heterodimer is ${\text{γ}}_{\text{p}17}$.

In our model, we did not explicitly include two copies of genes of homologous chromosomes in the wild-type embryos. We assume that bursts between two homologous chromosomes are perfectly correlated. In the deletion mutant, one chromosome copy is deleted. In our model, we reduce the maximum burst size ${\text{B}}_{\text{max}}$ by half to mimic the deletion mutant. Table S3 lists the parameters for wild-type and deletion mutants.

#### Simulations and noise measurement

All the biochemical reactions occur stochastically according to the propensities specified in Table S2. We numerically evolve the system’s stochastic dynamics using our custom C++ code in accordance with the Gillespie algorithm (Gillespie, 1976) (code is provided in Data S1 document). We generate many trajectories from the same initial condition. One trajectory represents the dynamics of a single cell. After the time, $T_{0}$, we store the values $m1_{n}$, and in the interval of $dt_{obs}$ (which is sufficiently smaller than the period of oscillation) for many time points up to time $T_{max}$. We calculate correlated and uncorrelated variability for a given time point data. Finally, we bin the correlated and uncorrelated variability for different time points according to the total *her* mRNA level ($\left\langle m1_{n}\right\rangle +\left\langle m7_{n}\right\rangle$) and compute the average value of variability at each bin.

## Data Availability

• The data reanalyzed in Figures 1 and 2 were previously published and deposited online as described in (Zinani et al., 2021), and are publicly available in the BioStudies database as of the date of publication. The accession number is listed in the key resources table. • Data in Figure 3 are newly generated according to the protocols described in (Zinani et al., 2021) and provided as Data S2 and S3. The raw data have been deposited at the BioStudies database, and are publicly available as of the date of publication. The accession number is listed in the key resources table. • C++ code is provided in Data S1 document. • Analyses are conducted in Python. The Python codes were previously made available online as described in (Zinani et al., 2021). • Any additional information required to reanalyze the data reported in this paper is available from the lead contact upon request.

## Supplemental information

Document S1. Figure S1 and Tables S1–S3

Data S1. C++ code running stochastic simulations is provided, related to Figure 2

Data S2. smFISH data obtained from her1ci301;her7hu2526 embryos is provided, related to Figure 3

Data S3. smFISH data obtained from her1b567/ci301;her7b567/hu2526 embryos is provided, related to Figure 3
