# Supplementary material for: Gene copy number and negative feedback differentially regulate transcriptional variability of segmentation clock genes
Source: iScience. 2022 Jun 11;25(7):104579. doi: 10.1016/j.isci.2022.104579 (PMC9250017; doi:10.1016/j.isci.2022.104579)
Supplement: Document S1. Figure S1 and Tables S1–S3 [file mmc1.pdf]

iScience, Volume *25*

Supplemental information

Gene copy number and negative feedback differentially regulate transcriptional variability of segmentation clock genes

Oriana Q.H. Zinani, Kemal Keseroğlu, Supravat Dey, Ahmet Ay, Abhyudai Singh, and Ertuğrul M. Özbudak

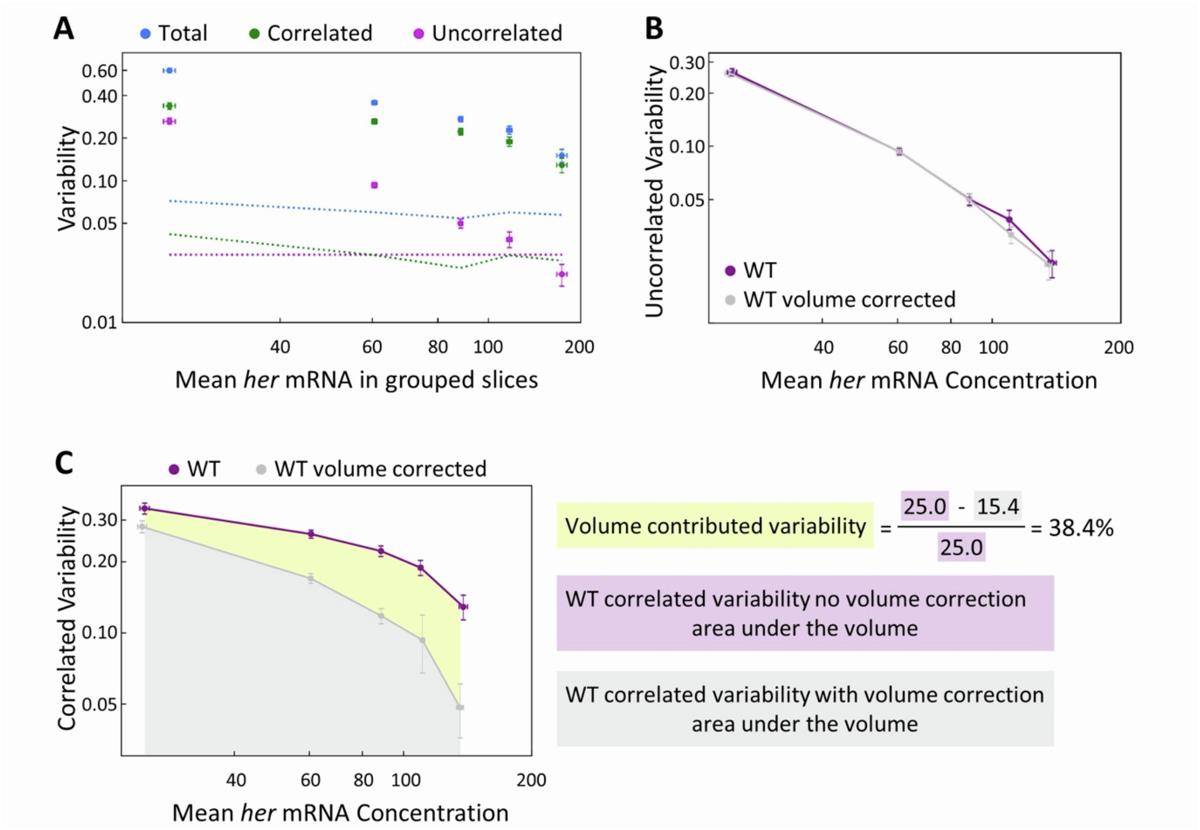

Figure S1. The contribution of volume-dependent heterogeneities on the correlated transcriptional variability of clock genes, Related to Figure 1.
(A) Transcriptional variability and mean RNA levels are grouped in five bins based on mean RNA levels. Correlated transcriptional variability is higher than uncorrelated one in all bins. The baseline variability due to measurement errors are plotted as dotted curves with the color-matched dashed lines as described in (Keskin et al., 2018). (B,C) The volume-independent transcriptional variability of two clock genes is measured by converting RNA counts to RNA concentration (per 4 pl, silver). By normalizing RNA counts to concentrations in 4 pl, the range of x-axis is kept constant between mRNA number counts (WT, purple) and mRNA concentration (silver). The uncorrelated variability overlapped in volume corrected and uncorrected analyses (B). The contribution of volume-dependent heterogeneities on the correlated transcriptional variability of clock genes is calculated by subtracting the area between the curves (C). Error bars are two standard errors.

Table S1: Notations for molecular species, Related to Figure 2 and STAR Methods.

Species counts	Symbol
Pre *her1* and *her7* mRNAs	$m1_0, m7_0$
Intermediate *her1* and *her7* mRNAs	$m1_j, m7_j$ for $j \in 1,2,..,n-1$
Mature or delayed *her1* and *her7* mRNAs	$m1_n, m7_n$
Pre (Cytoplasmic) Her proteins	$p1_0, p7_0$
Intermediate Her1 and Her7 proteins	$p1_j$ $p7_j$ for $j \in 1,2,..,n-1$
Mature (Nuclear) Her proteins	$p1_n, p7_n$
Her heterodimer	$p17$

Table S2: Biochemical reactions, Related to Figure 2 and STAR Methods.

**Reaction**	**Propensity**
mRNA Synthesis:	
Burst of *her1* mRNA: $\mathrm{m1_0 \rightarrow m1_0 + B_1}$	$\mathrm{k_{m1}}$
Burst of *her7* mRNA: $\mathrm{m7_0 \rightarrow m7_0 + B_7}$	$\mathrm{k_{m7}}$
Correlated bursts of *her1* and *her7* mRNAs: $\mathrm{m1_0 \rightarrow m1_0} + B,\ \mathrm{m7_0 \rightarrow m7_0} + B$	$\mathrm{k_{m17}}$
Transcriptional time delay:	
$\mathrm{m1_j \rightarrow m1_j - 1,\ m1_{j+1} \rightarrow m1_{j+1} + 1}$; for j $\in 0,1,2,..,\mathrm{n}-1$	$\mathrm{n}/\tau_\mathrm{m}$
$\mathrm{m7_j \rightarrow m7_j - 1,\ m7_{j+1} \rightarrow m7_{j+1} + 1}$; for j $\in 0,1,2,..,\mathrm{n}-1$	$\mathrm{n}/\tau_\mathrm{m}$
mRNA degradations:	
$\mathrm{m1_n \rightarrow m1_n - 1}$	$\gamma_\mathrm{m1}\,\mathrm{m1_n}$
$\mathrm{m7_n \rightarrow m7_n - 1}$	$\gamma_\mathrm{m7}\,\mathrm{m7_n}$
Protein synthesis:	
$\mathrm{p1_0 \rightarrow p1_0 + 1}$	$\mathrm{k_{p1}\,m1_n}$
$\mathrm{p7_0 \rightarrow p7_0 + 1}$	$\mathrm{k_{p7}\,m7_n}$
Translational time delay:	
$\mathrm{p1_j \rightarrow p1_j - 1,\ p1_{j+1} \rightarrow p1_{j+1} + 1}$; for j $\in 0,1,2,..,\mathrm{n}-1$	$\mathrm{n}/\tau_\mathrm{p}$
$\mathrm{p7_j \rightarrow p7_j - 1,\ p7_{j+1} \rightarrow p7_{j+1} + 1}$; for j $\in 0,1,2,..,\mathrm{n}-1$	$\mathrm{n}/\tau_\mathrm{p}$
Protein degradations:	
$\mathrm{p1_n \rightarrow p1_n - 1}$	$\gamma_\mathrm{p1}\,\mathrm{p1_n}$
$\mathrm{p7_n \rightarrow p7_n - 1}$	$\gamma_\mathrm{p7}\,\mathrm{p7_n}$
Dimer formation, dissociation, and degradation:	
$\mathrm{p1_n \rightarrow p1_n - 1,\ p7_n \rightarrow p7_n - 1,\ p17 \rightarrow p17 + 1}$	$\mathrm{k_b\,p1_n\ p7_n}$
$\mathrm{p1_n \rightarrow p1_n + 1,\ p7_n \rightarrow p7_n + 1,\ p17 \rightarrow p17 - 1}$	$\mathrm{k_u\,p17}$
$\mathrm{p17 \rightarrow p17 - 1}$	$\gamma_\mathrm{p17}\,\mathrm{p17}$

Table S3: Parameter values, Related to Figure 2 and STAR Methods.

Parameter	Value		Parameter	Value
k_m	4.0 min^{-1}		$\gamma_{m1} = \gamma_{m7}$	0.4 min^{-1}
α	1.0		$\gamma_{p1} = \gamma_{p7}$	0.3 min^{-1}
B_{max} for 2 (or 1) chromosomes	10 (5)		γ_{p17}	0.3 min^{-1}
k_{p1}=k_{p7}	4.5 min^{-1}		τ_m	9.0 min
k_b	1.0 min^{-1}		τ_p	1.0 min
k_u	1.0 min^{-1}		n	10
pd_{crit}	100.0		T_0	100 min
dt_{obs}	0.1 min		T_{max}	500 min
